# Supplementary material for: Estimating flowering transition dates from status-based phenological observations: a test of methods
Source: PeerJ. 2019 Sep 24;7:e7720. doi: 10.7717/peerj.7720 (PMC6764363; doi:10.7717/peerj.7720)
Supplement: Supplemental Information 1 [file peerj-07-7720-s001.pdf]

Estimating flowering transition dates from status-based phenological observations: a test of methods

Shawn D. Taylor

Supplemental Images S1-S5

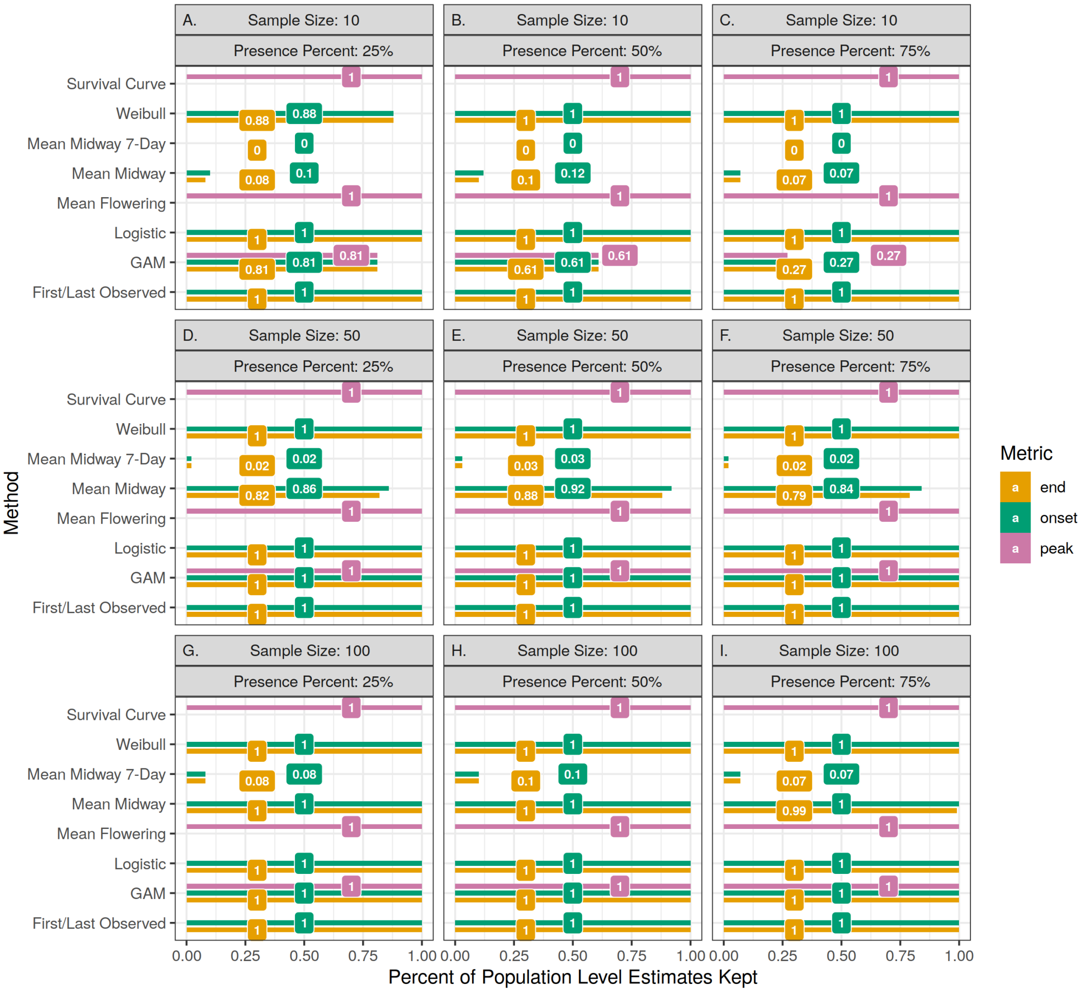

Figure S1: For all population level analysis, the proportion of estimates which were usable for each estimator method. Randomly drawn sets of observations may not be usable due to filtering (ie. requiring an absence observation within 7 days of a presence observation) or due to lack of convergence in the models.

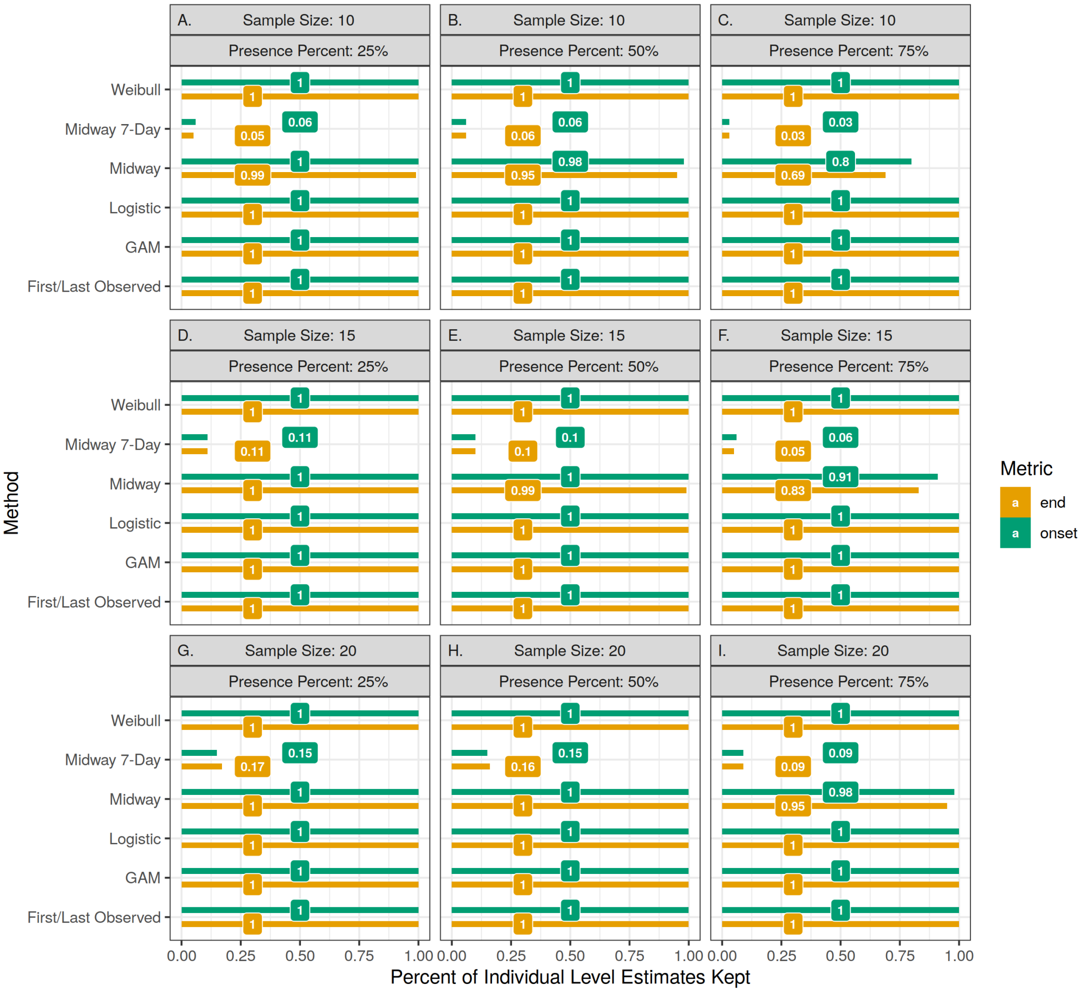

Figure S2: As in Figure S1, but for all individual level analysis.

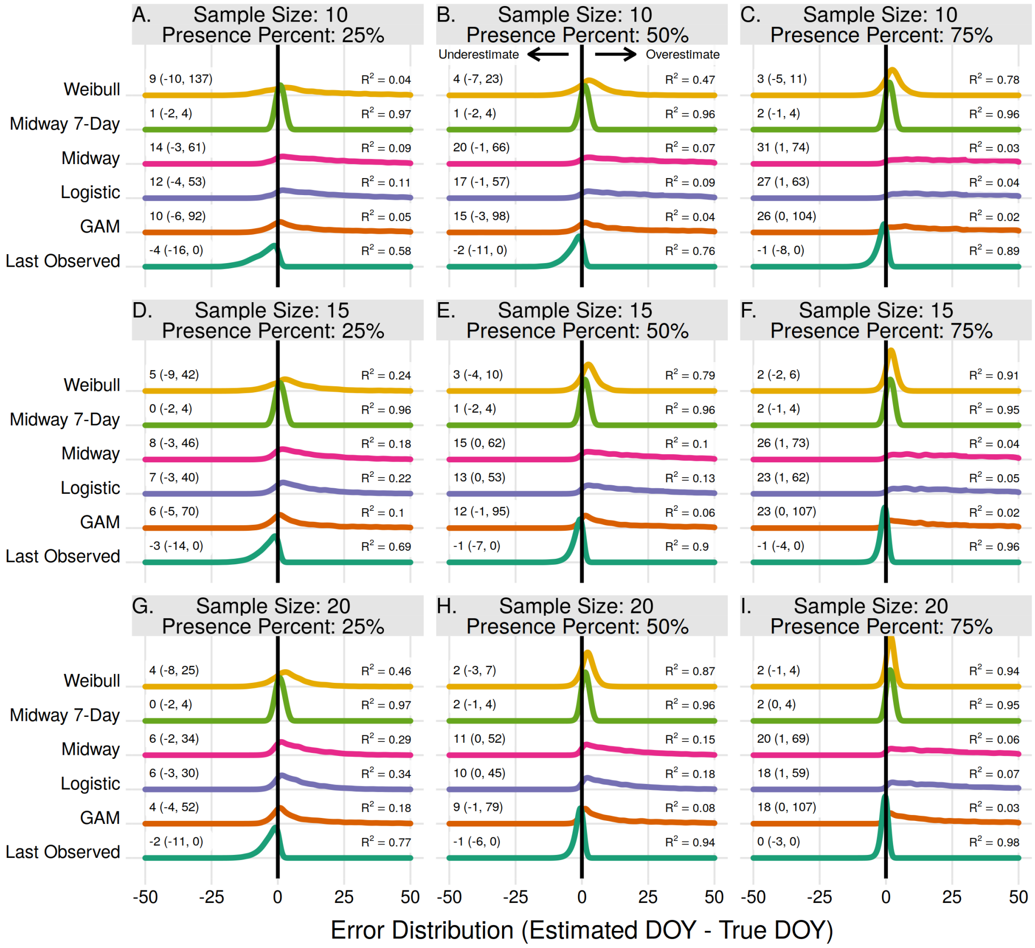

Figure S3: The error distribution of all estimators for individual flowering end. Text values represent the median error and the 95% quantile range in parenthesis.

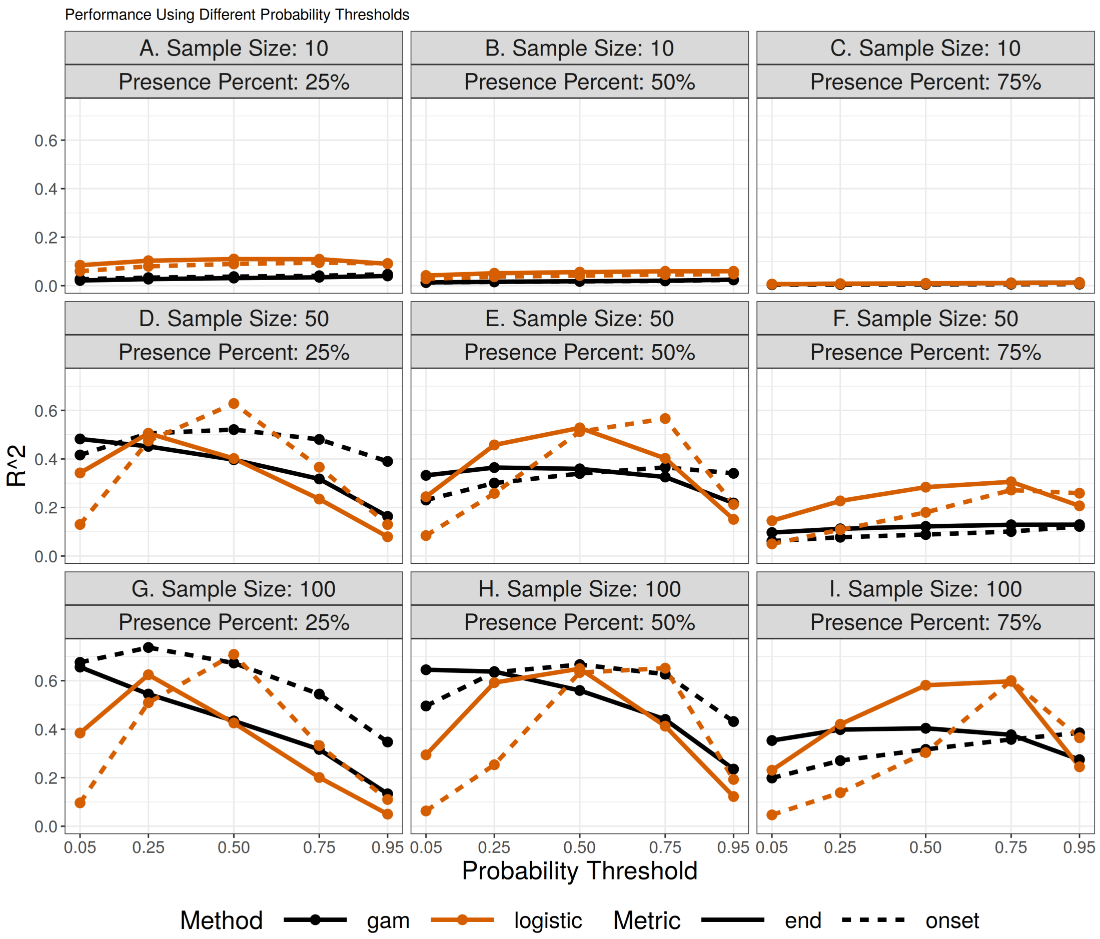

Figure S4: The R^2 for the GAM (black) and Logistic (red) methods in all scenarios and using a range of probability thresholds. Solid lines indicate the value for flowering end, while dashed lines indicate flowering onset. Each threshold was evaluated fully within the Monte Carlo analysis of the population level estimates.

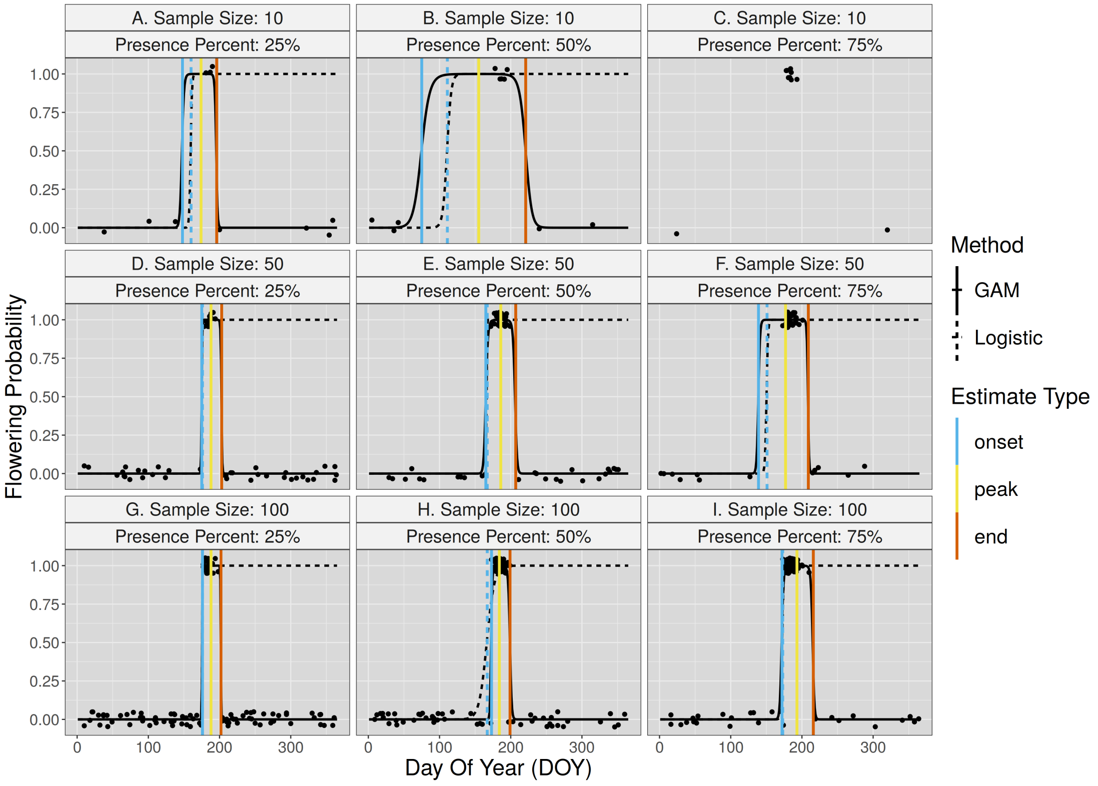

Figure S5: Visualization of a GAM and Logistic estimates of a single Monte Carlo run from the population level analysis. Points represent randomly sampled observations of flowering presence (1) and absence (0). Note the points are jittered slightly on the y-axis for clarity. The black lines represent the modelled probability of flowering for the full year for both GAM (solid) and Logistic (dotted) methods. Vertical color lines represent estimates from both GAM (solid) and Logistic (dotted) methods using a probability threshold of 0.50 for all cases except for the GAM peak estimate, which uses the maximum probability. Estimates for a sample size of 10 and percent yes of 0.75 were not possible due to the models failing to converge.

Note how as the proportion of presence observations increases, gaps in the absence data tend to become larger, resulting in probability curves which tend to underestimate flowering onset.
